# Synergistic effects of 5-fluorouracil in combination with salinomycin promoted ferroptosis via inhibiting SLC7A11/GPX4 in colorectal cancer

**DOI:** 10.3389/fonc.2025.1558290

**Published:** 2025-06-12

**Authors:** Can Wang, Junyang Wang, Fei Xing, Ning Liu, Xudong Wang

**Affiliations:** ^1^ Department of Gastrointestinal Surgery, The Second Hospital of Jilin University, Changchun, Jilin, China; ^2^ Central Lab, The Second Hospital of Jilin University, Changchun, Jilin, China

**Keywords:** salinomycin, 5-fluorouracil, drug sensitivity, ferroptosis, colorectal cancer

## Abstract

**Purpose:**

5-Fluorouracil (5-FU) resistance is considered to be a possible reason for the failure of conventional drug treatment of colorectal cancer (CRC). Recently, salinomycin (SAL), as a selective inhibitor of cancer stem cells (CSCs), has been used to sensitize and attenuate a variety of solid tumor chemotherapy drugs. In our study, our goal was to combine SAL with 5-FU to explore not only whether there is an increase in sensitivity of CRC to 5-FU but also the molecular mechanism involved in enhancing 5-FU sensitivity and promoting tumor cell chemotherapeutic death.

**Methods:**

ComboSyn software was used to study whether dual drug combinations synergistically promote each other and their dosage. CCK8, EdU, and Annexin V/PI assays were used to study the cell proliferation and apoptosis of SW480 and HCT116 cells in response to SAL single-drug and dual-drug co-treatment. Cell cycle staining was used to assess cycle arrest. Wound healing and migration and invasion experiments were used to identify changes in migration and invasion capabilities under the influence of different drugs. Transcriptome sequencing is used to explore the molecular mechanisms of drugs. Reactive oxygen species (ROS) fluorescence staining and malondialdehyde (MDA) level measurement were used to confirm the changes in ferroptosis levels of SW480 and HCT116 cells after drug treatment. Nude xenograft mice were used to detect antitumor *in vivo*. Changes in the protein level expression of ferroptosis GPX4 and SLC7A11 were also determined in the treated cells.

**Results:**

SAL alone and in combination with 5-FU were found to significantly increase cell mortality and apoptosis. At the same time, our results show that the combination of SAL and 5-FU not only inhibits the proliferation, migration, and invasion of CRC cell lines *in vivo* and *in vitro*, but also promotes ferroptosis of CRC cell lines by downregulating the expression of GPX4 and SLC7A11. It may provide more and novel solutions and treatment perspectives for 5-FU or other drug-resistant chemotherapy strategies for patients with CRC.

**Conclusions:**

SAL inhibits CRC, whose effect is achieved by reducing GPX4 and SLC7A11 protein levels to mediate ferroptosis activation in collaboration with 5-FU.

## Introduction

Colorectal cancer (CRC) remains a major global health challenge and is one of the leading causes of cancer-related deaths recently (1, 2). The field of CRC treatment has undergone dramatic changes in the past few decades, especially in the field of cancer chemotherapy. Despite this, chemotherapy based on 5-fluorouracil (5-FU), as a first-line treatment for advanced CRC, has a response rate of only 10%–15% (3, 4). Research on the sensitization and attenuation mechanism of 5-FU has become a major challenge in clinical treatment. Salinomycin (SAL) has emerged as a promising agent in the treatment of multiple cancers, particularly in its ability to enhance the efficacy of conventional and novel chemotherapeutics like doxorubicin (5), SN38 (an active metabolite of irinotecan) (6), gefitinib (7), gemcitabine (8), and cisplatin (9). To date, SAL and its analogs [including SAL-isohydroxamic acid conjugates (10), C20-O-alkyl/benzyl oxime derivatives (11), C20-O-acylated analog of SAL (12), and SAL nanocrystals (13)] are known to selectively target cancer stem cells (CSCs), which are often responsible for tumor initiation, metastasis, and resistance to chemotherapy (6, 12, 13). Even though SAL has great potential as a single drug to participate in the treatment of various cancers, objectively speaking, its value as a single drug needs further research and observation, and it may be more urgent to serve an auxiliary role for various mainstream chemotherapy drugs in increasing sensitivity and reducing resistance (14).

Our research aims to figure out that by reducing the population of CSCs, SAL may enhance the sensitivity of CRC cells to 5-FU, which primarily targets rapidly dividing cells but may be less effective against CSCs that exhibit a quiescent phenotype. We will attempt to observe and accurately describe the synergistic effect of SAL and 5-FU.

## Materials and methods

### Cell culture and reagents

The human CRC cell lines SW480 and HCT116 were purchased respectively from Applied Biological Materials Inc. (abm, Shanghai, China) and Haixing Biosciences Biotechnology (Jiangsu, China), supplemented with 10% fetal bovine serum (FBS, Procell, Wuhan, China) and 1% penicillin–streptomycin solution (P/S, 100×, Procell, Wuhan, China). With Dulbecco’s Modified Eagle Medium (DMEM, Procell, Wuhan, China), cells were cultured at 37°C in a 5% CO_2_ incubator under regulated conditions. The pharmaceutical powders of SAL (SJ-MA0092) and 5-FU (SJ-MX0076) were obtained from SparkJade Biotechnology Co., Ltd. (Shandong, China) and respectively prepared into 10 mM/mL solvents using dimethyl sulfoxide (DMSO, GC203006, Servicebio, Wuhan, China), stored at −20°C. The antibodies against SLC7A11 (A2413, 1:1,000) and GPX4 (A11243, 1:2,000) were obtained from ABclonal Technology (Wuhan, China) with primary antibody dilution buffer (P0023A, Beyotime, Shanghai, China). Antibodies against GAPDH (1:10,000) and β-actin (1:10,000) were obtained from Proteintech Group, Inc. (Wuhan, China).

### CCK8 cell proliferation experiment and determination of drug synergistic concentration

The cell lines with good cell growth status were diluted to 2 × 10^3^/well and then added into a 96-well plate, surrounded by 100 μL of PBS to replace evaporation to prevent cell edge effects and culturing overnight. The different drug gradients for SAL single drug (2.5, 5, 10, 20, 40, and 80 μM) and 5-FU single drug (0, 5, 10, 20, 40, 80, 160, and 320 μM) for 24, 48, and 72 h culture were used to evaluate drug IC_50_ value according to the instructions of the CCK-8 kit (Invigentech, California, USA). Then, the 96-well plate was incubated simultaneously at 37°C for 2 h and the absorbance value was measured at a wavelength of 450 nm with a Varioskan flash multimode reader (Thermo Scientific, USA). Subsequently, a string of drug concentrations lower than the 72-h IC_50_ in the study were selected to conduct 15 different combinations by addressing CCK8 experiments to observe the cytotoxic effect, and the ComboSyn software (https://www.combosyn.com/, PD Science, LLC., 27 June 2023) was used to evaluate the effect of different double-drug combinations. It is based on Chou-Talalay methods (15, 16) using the combination index (CI) such that the software could provide users with the results of whether two or more drugs with different ingredients can have synergistic effects (CI < 1), additive effects (CI = 1), or antagonistic effects (CI > 1), shown as the Fa-CI figure: The dotted line CI = 1 in the Fa-CI diagram is considered to have an additive effect, while the points above the dotted line indicate that the two drugs are resistant to each other and those below the dotted line indicate a synergistic effect. Select the appropriate concentration according to the minimum dose principle that produces a synergistic effect of the drug and measure the IC_50_ again. The dose reduction index (DRI) for HCT116 and SW480 cell lines treated with salinomycin and 5-FU is presented in **Supplementary Table 2**.

### EdU cell proliferation assay

Seed 3 × 10^4^ cells per well into a confocal dish and culture overnight until the cells adhere to the normal growth state, treated with 0.1% DMSO, 2.5 μM SAL, 5 μM 5-FU, and 2.5 μM SAL + 5 μM 5-FU for 20 h, respectively. EdU (10 mM) was mixed with 1:500 medium into a final concentration of 20 μM 2× EdU working solution, according to the Beyotime (Beyotime, China) EdU instruction manual, fixed with 4% paraformaldehyde at room temperature for 15 min, permeated with PBS with 0.3% Triton X-100, and washed, and the Click reaction solution was prepared and incubated with DAPI for 30 min. DAPI is excited at a wavelength of 405 nm, while EdU is excited at a wavelength of 555 nm, and run on an FV1000 Confocal Laser Scanning Biological Microscope (Olympus, Japan) for detection.

### Cell cycle and apoptosis staining

The logarithmic phase cells in good growth condition were passaged to 6-cm medium dishes and treated with 0.1% DMSO, 2.5 μM SAL, 5 μM 5-FU, and 2.5 μM SAL + 5 μM 5-FU for 20 h, respectively. For the cell cycle, pellet cells were fixed in 70% pre-cooled ethanol at 4°C overnight, washed, and resuspended. Next, 25 μL of PI and 10 μL of RNase A/0.5 mL staining working solution were added to each sample, incubated at room temperature in the dark for 30 min, and then a flow cytometry analysis tube was used to detect sample fluorescence at 488 nm wavelength. For cell apoptosis, the cells were digested and precipitated together with the supernatant in the culture medium, a total of 5 × 10^5^ cells were resuspended, and a staining solution was prepared for each sample with 500 μL of 1× Annexin V Binding Buffer, 5 μL of Annexin V-FITC, and 10 μL of PI, which was incubated at room temperature for 20 min for detection purposes.

### Colony formation assay

Cells were inoculated at 2 × 10^3^/well, and after 24 h of adhesion, they were treated with 0.1% DMSO, 2.5 μM SAL, 5 μM 5-FU, and 2.5 μM SAL +5 μM 5-FU for 20 h, and then the medium was replaced and cultured for 14 days. The medium was changed every 3 days, the cell status was observed, and the culture was terminated within 14 days when the number of clones in the minimum well is greater than 50. The medium was fixed with 1.5 mL/well of 4% formaldehyde solution at room temperature for 30 min, then crystal violet dye solution was added for staining at room temperature for 20 min, followed by washing and taking photos.

### Wound healing assay

Plate 1 × 10^5^ cells/well in a six-well plate, and after adhering to the wall, use a 200-μL pipette tip along the ruler to scratch on the cell plane perpendicular to the transverse diameter of the six-well plate. During the process, try not to continue the scratching process and do not tilt the pipette tip. Each hole was scratched three times vertically with an interval of 0.5 or 1 cm, and the white field images were taken using a fluorescence microscope at approximately the same position at 0 and 48 h, respectively. ImageJ (https://imagej.net/ij/) for Windows was downloaded and then used to measure the area between the scratches and perform statistical analysis.

### Transwell migration and invasion assay

For the migration experiment, cells were starved for 24 h in serum-free DMEM in advance, cell suspension was prepared at 5 × 10^4^ cells/mL, and the following drugs were added: 0.1% DMSO, 2.5 μM SAL, 5 μM 5-FU, and 2.5 μM SAL + 5 μM 5-FU. After the basement membrane was hydrated with culture medium in the upper chamber of the Transwell plate for 30 min, 600 μL of 15% fetal calf serum and DMEM complete medium was added to the lower chamber, and then the drug-treated cell suspension was added to the upper chamber at a volume of 100 μL per well, and the cell suspension was thoroughly mixed crosswise and cultured for 48 h. For the invasion experiment, after starving cells in serum-free DMEM medium for 24 h, the cell suspension was prepared at 2 × 10^4^ cells/mL, and the following drugs were added: 0.1% DMSO, 2.5 μM SAL, 5 μM 5-FU, and 2.5 μM SAL + 5 μM 5-FU. The Matrigel was melted at 4°C in advance, using the 4°C pre-cooled pipette tip to dilute the gel with serum-free DMEM medium at a ratio of 1:10, then the gel solution was added to the hydrated Transwell chamber and placed in a CO_2_ incubator for 2 h. Likewise, 600 μL of 15% FBS DMEM complete culture medium was added to the lower chamber and 100 μL of drug-treated cell suspension was added to the upper chamber; they were mixed thoroughly and placed in an incubator for 72 h. After the above culture, the mixture was fixed with 4% paraformaldehyde at room temperature for 30 min. While washing, add crystal violet for staining at room temperature for 30 min and use a disposable cotton swab to gently wipe the inner membrane of the upper chamber to remove the nonspecifically attached dye, and then take pictures under a white field fluorescence microscope.

### Tumorigenesis experiments in nude mice *in vivo*

The SPF-grade constant-temperature animal breeding center was used for raising mice and was given a suitable environment in accordance with experimental animal ethics. Before the start of the experiment, the 24 mice were kept stable for a week; no obvious stress responses or other conditions were observed. The cells were collected and used in serum-free DMEM to make a cell suspension of 2 × 10^7^ cells/mL, and kept on ice for later use. Tumors were formed by subcutaneous injection into the groin of mice. Each mouse was injected with 100 μL of suspension using an insulin syringe. Before injection, each mouse was anesthetized by intraperitoneal injection of 0.3% sodium pentobarbital 0.2 mL/10 g (final concentration approximately 40 mg/kg). Mice were divided into groups (each group *n* = 5, reserving 4 to avoid unexpected situations): 0.1% DMSO control, 5 mg/kg SAL, 25 mg/kg 5-FU, and SAL + 5-FU. The mice were injected intraperitoneally every other day in the right groin for a total of seven times. SAL was prepared into a 1 mg/mL solution: 10 mg of SAL is dissolved in 200 µL of DMSO to prepare a 50 mg/mL stock solution. Then, 28 µL of the stock solution is diluted with 1,372 µL of PBS to achieve a working concentration of 1 mg/mL to ensure that the DMSO concentration is lower than 0.1%, administered at a dose of 5 mg/kg, as previously described. For 5-FU, 60 mg is dissolved in 12 mL of PBS to prepare a 5 mg/mL working solution, which is then administered at a dose of 25 mg/kg for injection. When the maximum diameter of tumors grown in mice in the 0.1% DMSO control group within 14 days is 1 cm measured with a vernier caliper, the experiment is terminated, and the mice are euthanized after anesthesia with CO_2_, and the corresponding tumors, organs, and blood are collected for testing or fixed and embedded.

### Tissue of tumors and cytokine ELISA detection

The maximum diameter and weight of mouse tumors were measured to evaluate the tumor killing effect of different drug treatment groups. Collect mouse serum and detect the concentrations of tumor necrosis factor-α (TNF-α), interleukin-10 (IL-10), and vascular endothelial growth factor (VEGF) cytokines for supplementary assessment according to their respective instructions.

### HE staining

The tumor tissues from the nude mice were collected and sent to Sevier Biotechnology Co., Ltd. for histopathological sectioning (refer to the company's paraffin embedding protocol). Important organs of mice, such as heart, liver, spleen, lungs, and kidneys, were used for hematoxylin and eosin (HE) staining to evaluate the effects of different drug treatments on these organs.

### Transcriptomic sequencing and gene set assessment

Sequencing services were provided by Meiji Biotechnology and implemented in the following ways: Each group of 2 × 10^6^ cells was used for eukaryotic mRNA sequencing, total RNA was provided from the cell samples, RNA concentration and purity were detected using Nanodrop2000, and then a whole-genome kit was used for RNA library construction. Use Oligo (dT) magnetic beads with polyA for base complementary pairing to isolate and enrich the mRNA. Then, add fragmentation buffer to randomly fragment the mRNA. Small fragments (300 bp) were sorted by magnetic beads and reverse transcribed into double fragments using a reverse transcription kit. Strand cDNA was filled with End Repair Mix and connected to the adapter. After the above processing, the Illumina platform was used, consisting of the following: (i) library enrichment, PCR amplification cycles; (ii) 2% agarose recovery; (iii) TBS380 (Picogreen) machine; (iv) cBot bridge PCR amplification to generate clusters; and (v) PE library, read length 2×150 bp, sequenced. The original sequencing data were processed by the Meji Bioinformatics platform to obtain the expression matrix. In view of the complexity of this grouping, it is difficult for conventional difference analysis to meet the requirements. In order to highlight the regulation of dual-drug combination pathways, we used GSVA and GSEABase packages to analyze the Hallmark signaling pathway and the author collected and sorted 34 Kyoto Encyclopedia of Genes and Genomes (KEGG) (https://www.genome.jp/kegg/) signaling pathways; 18 human cell death pathways were determined by the pathway scores in each group of samples. After the signal pathway score was passed through *Z*-score, the ggplot2 package was used to display the activation/inhibition of the signal pathway between each group.

### Cell transmission electron microscopy

The cells were administered in groups of 0.1% DMSO, 2.5 μM SAL, 5 μM 5-FU, and 2.5 μM SAL + 5 μM 5-FU for 20 h. The cells were collected by centrifugation, added with an electron microscope fixative, and submitted to the company, Liaoning Jijia Biotechnology Co., Ltd; refer to the company guide for specific steps.

### Transmission electron microscopy

The cell TEM (transmission electron microscopy) was commissioned to Liaoning Jijia Biotechnology Co., Ltd. In short, the conventional electron microscopy processing protocol: drug treatment - embedding - dehydration - sectioning - observing the characteristic changes of mitochondria within cells to determine whether the features of ferroptosis appear.

### ROS fluorescence staining

The cells were administered in groups of 0.1% DMSO, 2.5 μM SAL, 5 μM 5-FU, and 2.5 μM SAL + 5 μM 5-FU for 20 h. DCFH-DA was diluted according to the ratio of 1:1,000, while DAPI was added in the same ratio and incubated at 37°C in the dark for 20 min. Cells were washed three times with PBS and observed under a fluorescence microscope, and ImageJ software was used to quantitatively carry out intDen/cell number analysis.

### MDA concentration determination

The cells were administered in groups of 0.1% DMSO, 2.5 μM SAL, 5 μM 5-FU, 2.5 μM SAL + 5 μM 5-FU for 20 h. Discard the supernatant; collect the cells with a cell scraper; add reagent 5; ultrasonically homogenize and crush the cells; configure the application solution and chromogenic solution according to the instructions (A003-4-1, Nanjin Jiancheng, China); set up two blank tubes, standard tubes, and measurement tubes; vortex and mix the cells and then bathe them in 95°C water for 40 min. After completion, cool to room temperature with running water, centrifuge at 4,000*g* for 10 min, add 250 μL of each well into a 96-well plate, measure the absorbance at a wavelength of 530 nm, and simultaneously measure the protein concentration of each EP tube with BCA in the water bath. Malondialdehyde (MDA) (nmol/mgprot) = (measured OD value − blank OD value)/(standard OD value − blank OD value) *10 nmol/mL/sample protein concentration.

### Total protein extraction and western blot electrophoresis detection

After treatment for 20 h according to the above-mentioned drug groups, the cells were placed on ice and treated with PMSF (1:100, Beyotime, Shanghai, China), protease inhibitor (1:100, absin, Shanghai, China), phosphatase inhibitor (1:100, absin, Shanghai, China), and nuclease (1:1,000, Beyotime, Shanghai, China). Prepare the cell lysis solution proportionally. Add the lysis solution at a dosage of 300 μL lysis solution/6 cm medium dish or 100 μL lysis solution/six-well plate per well. Use a cell scraper to collect the cells into an EP tube and lyse them on ice for 30 min at 4°C. Centrifuge at 14,000g and take the supernatant and quantify according to the BCA protein quantification kit (Epizyme, Shanghai, China). After protein quantification, adjust the protein concentration to 15 μg/10 μL with 5× loading buffer (Epizyme, Shanghai, China) and lysis buffer, and place the cells in a 100°C water bath or metal bath for 8 min, cool to room temperature, then aliquot and store at −20°C. Use an Epizyme SDS-PAGE gel preparation kit to prepare 10% or 15% electrophoresis gel in a sandwich structure and put it into the electrophoresis instrument, then transfer the membrane, block with milk (Servicebio, Wuhan), and incubate the primary and secondary antibodies.

### Statistical analysis

The statistical data in this study are expressed as mean ± standard deviation. For transcriptome gene set ssGSEA score differences, the Mann–Whitney test was used to evaluate the differences between different groups. The difference test between different groups used GraphPad Prism 9 to perform one-way analysis of variance and Sidak multiple group test to calculate the *p*-value between the two groups among all remaining parts. *p*-value <0.05 was considered to be statistically different.

## Results

### Combination of SAL and 5-FU inhibited the viability of HCT116 and SW480 cell lines

**Figure 1:**
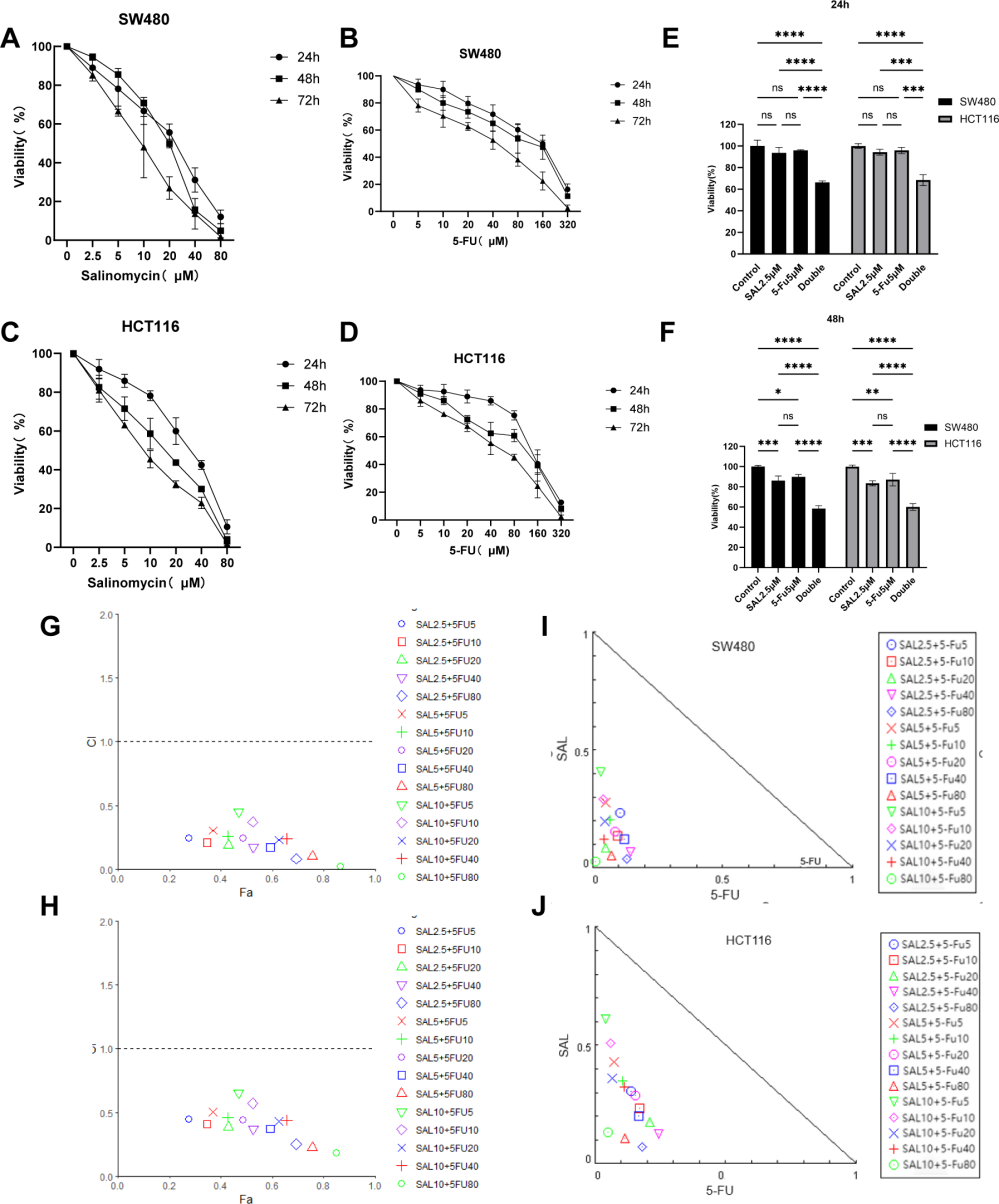
**(A, B)** The SW480 cell line is treated with SAL and 5-FU, respectively, to inhibit cell proliferation. **(C, D)** The HCT116 cell line is treated with SAL and 5-FU to inhibit cell proliferation, respectively. **(E)** The combination of 2.5 μM SAL and 5 μM 5-FU significantly inhibited the cell viability of SW480 cell line for 24 and 48 h **(F)** The combination of 2.5 μM SAL and 5 μM 5-FU significantly inhibited the cell viability of SW480 cell line for 24 and 48 h **(G, H)** Fa-CI diagrams of different combinations of SW480 and HCT116 cell lines are shown, respectively (CI < 1 means that the drug combination has a synergistic effect). **(I, J)** The SAL and 5-FU isobolograms of SW480 and HCT116 are shown, respectively (locating inside the triangle has a synergistic effect). Compared with the control group: * or ns, *p* < 0.05; **p < 0.01; ***p < 0.001; ****p < 0.0001.

First, we tested the effects of SAL and 5-FU on CRC cells in monotherapy and combination therapy through the CCK8 assay, treated with different doses of the drug for 24, 48, and 72 h (**Figures 1A–D**). We found that SAL and 5-FU significantly inhibited the viability of SW480 and HCT-116 cells in a dose- and time-dependent manner. IC_50_ for each drug individually and the dose of counterparts were selected based on the cytotoxic effects when added alone. For SAL, the 72-h IC_50_ value for the SW480 cell line is 14.97 μM and that for the HCT116 cell line is 9.306 μM. For 5-FU, the IC_50_ value for the SW480 cell line is 35.45 μM and that for the HCT116 cell line is 45.94 μM (**Supplementary Table 1**). The drug concentrations below the 72-h IC_50_ and the concentration of SAL and 5-FU co-treated were respectively chosen by 15 different combinations (SAL for 2.5, 5, and 10 μM and 5-FU for 5, 10, 20, 40, and 80 μM). For any drug combination to exhibit synergistic effects, it must meet the following criteria: (1) The CI on the Fa-CI plot must lie below the line where CI = 1.0; (2) the SAL-5-FU isobologram must be positioned below the diagonal line. Our results indicate that the drug combinations mentioned above exhibit synergistic effects in both SW480 and HCT116 cells (**Figures 1G–J**). The combined effect of 2.5 μM SAL and 5 μM 5-FU was confirmed for the further study, based on the principle of selecting the minimum concentrations that induce synergy and could effectively inhibit cell viability at 24 and 48 h (**Figures 1E, F**) for both SW480 (*p* < 0.0001) and HCT116 (*p* < 0.0001), when the combination was significantly stronger than 2.5 μM SAL or 5 μM 5-FU alone (*p* < 0.0001).

### Drug incorporation significantly inhibited proliferation, invasion, and migration and promoted apoptosis of colorectal cancer cell lines

**Figure 2:**
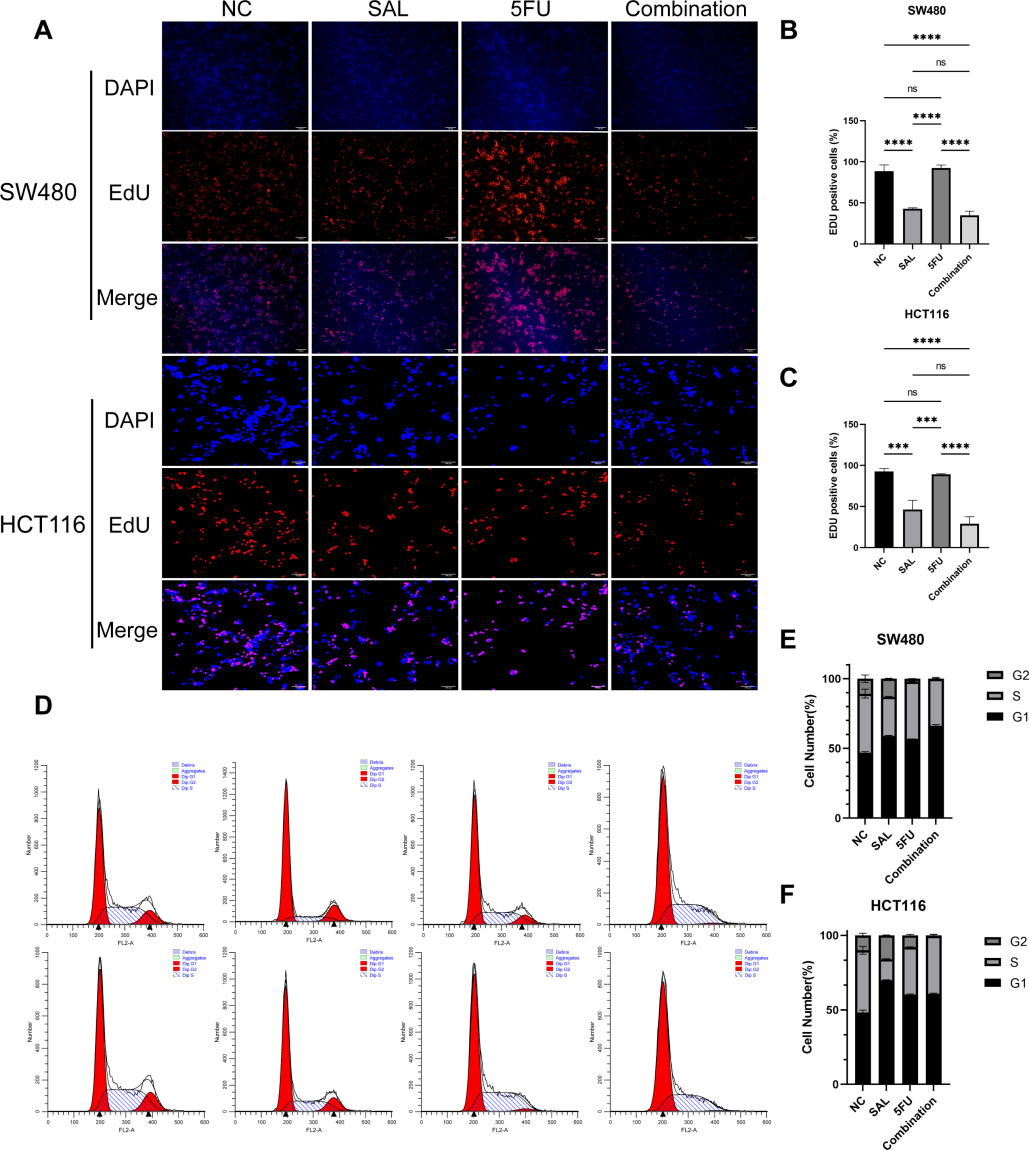
**(A)** EdU staining results of SW480 and HCT116 cell lines after treatment with different drugs for 20 h. **(B, C)** Statistical analysis of EdU staining results of SW480 and HCT116 cell lines after administration. **(D)** Cell cycle staining results of SW480 and HCT116 cell lines after treatment with different drugs for 20 h. **(E, F)** Statistical analysis of cell cycle staining results of SW480 and HCT116 cell lines after administration. Compared with the control group: ns, *p* < 0.05; ***p < 0.001; ****p < 0.0001.

**Figure 3:**
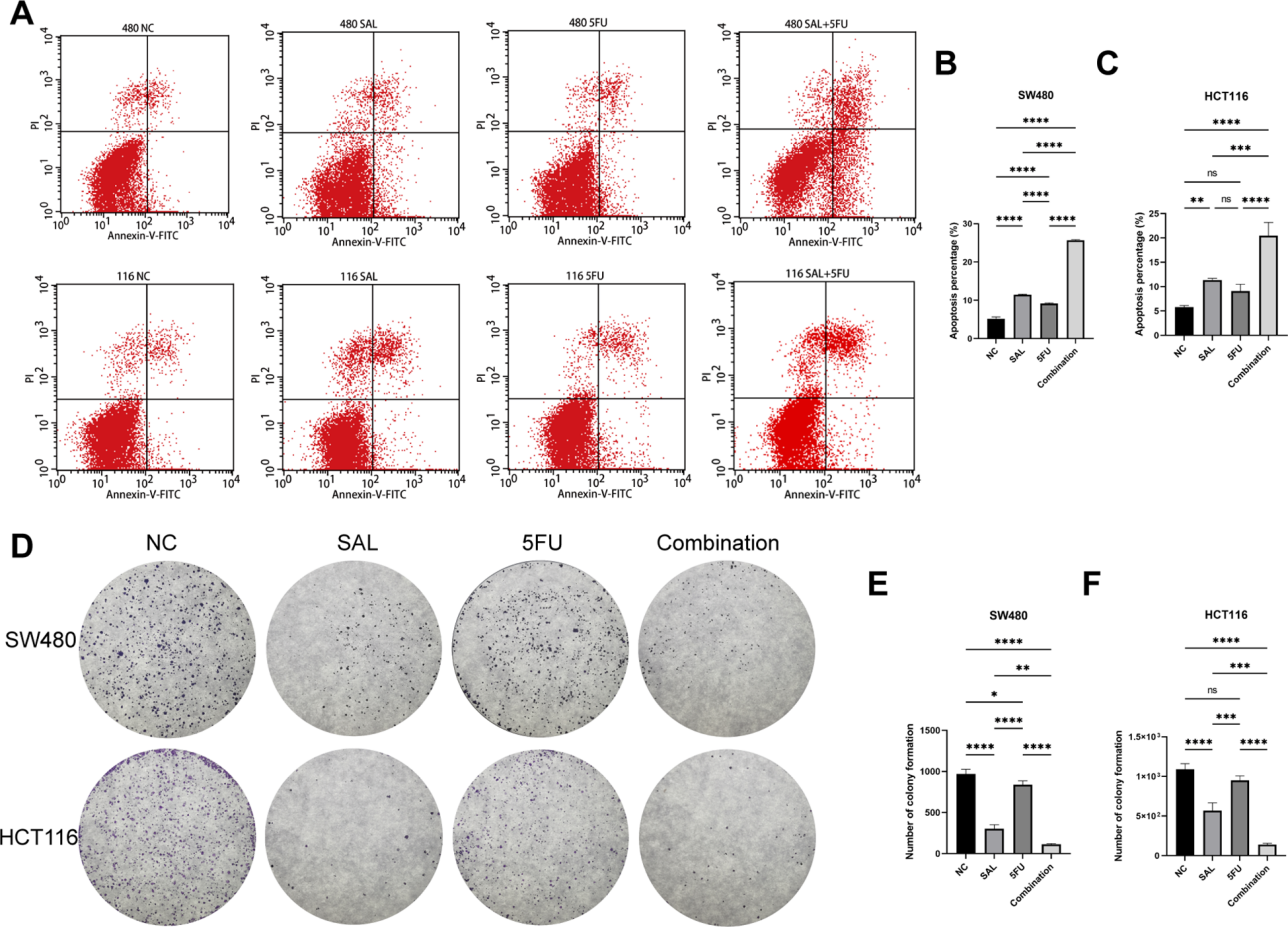
**(A)** Annexin-V-FITC/PI apoptosis staining results of SW480 and HCT116 cell lines after treatment with different drugs for 20 h. **(B, C)** Statistical analysis of apoptosis staining results of SW480 and HCT116 cell lines after administration. **(D)** Crystal violet staining results of colony formation assay on HCT116 and SW480 cell lines after different treatments. **(E, F)** Statistical analysis of colony formation assay results of SW480 and HCT116 cell lines after administration. Compared with the control group: * or ns, *p* < 0.05; **p < 0.01; ***p < 0.001; ****p < 0.0001.

**Figure 4:**
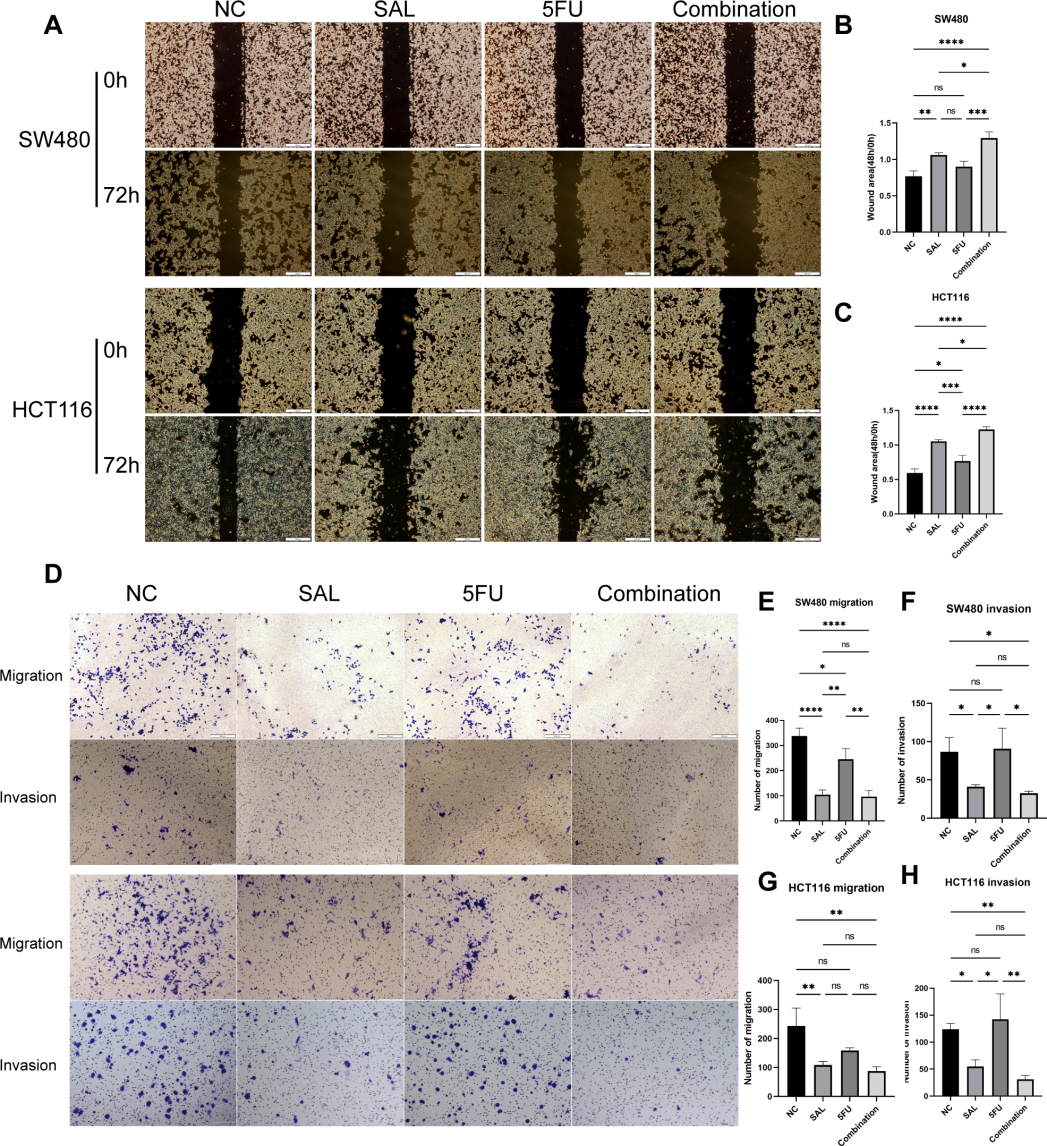
**(A)** Results of wound healing assays on HCT116 and SW480 cell lines after different administration treatments. **(B, C)** Statistical analysis of wound healing assays results of SW480 and HCT116 cell lines after administration. **(D)** Results of migration and invasion assays on HCT116 and SW480 cell lines after different administration treatments. **(E–H)** Statistical analysis of migration and invasion assay results of SW480 and HCT116 cell lines after administration, respectively. Compared with the control group: * or ns, *p* < 0.05; **p < 0.01; ***p < 0.001; ****p < 0.0001.

EdU staining was used to evaluate the proliferative potential of cell lines, and our results confirmed that drug combination can significantly inhibit cell proliferation of both SW480 (*p* < 0.0001) and HCT116 (*p* < 0.0001) compared to even monotreatment after a 24-h period of addressing (**Figures 2A–C**). As expected, G1/S phase arrest occurred in the 5-FU group; surprisingly, almost no cells in the G2 phase were found in the combination treatment group, and our results prove that the combination of SAL and 5-FU can promote CRC cell cycle arrest (**Figures 2D–F**, **Supplementary Figure S3**). Similarly, Annexin-V/PI staining also assisted in confirming the above conclusion that, at this concentration, adding 2.5 μM SAL can significantly promote cell apoptosis alone (SW480, *p* < 0.0001; HCT116, *p* < 0.01) and the same effect can be detected in the group of 2.5 μM SAL + 5-FU of both SW480 (*p* < 0.0001) and HCT116 (*p* < 0.0001) (**Figures 3A–C**). The colony formation assay showed that in the SW480 cell line, the use of 2.5 μM SAL as well as the dual-drug combination significantly inhibited the cloning ability of tumor cells; for the blank counterpart, similar situations exist (for SW480 SAL vs. NC, *p* < 0.0001 and Combination vs. NC, *p* < 0.0001; for the HCT116 cell line SAL vs. NC and Combination vs. NC, *p* < 0.0001; **Figures 3D–F**). Wound healing assays demonstrate the ability of dual drugs to synergistically kill cells and inhibit migration (*p* < 0.01 for SW480 SAL vs. NC and *p* < 0.0001 for Combination vs. NC; for HCT116: *p* < 0.001 for SAL vs. NC and Combination vs. NC, **Figures 4A–C**). The Transwell assay demonstrated that the integration of SAL and 5-FU could significantly alleviate migration (for SW480: *p* < 0.001 for SAL vs. NC and Combination vs. NC, *p* < 0.01 for Combination vs. 5-FU; for HCT116: *p* < 0.01 for SAL and Combination vs. NC) and invasion abilities testified by being covered with 3% Matrigel (for SW480: *p* < 0.05 for SAL vs. NC and Combination vs. NC, Combination vs. 5-FU; while *p* < 0.05 for SAL vs. NC and *p* < 0.01 for Combination vs. NC and 5-FU for HCT116, **Figures 4D–H**). In short, our work found that drug combination can synergistically regulate cell growth, migration, and invasion capabilities.

### The combination therapy triggered significantly and synergistically cytotoxic effects *in vivo*

**Figure 5:**
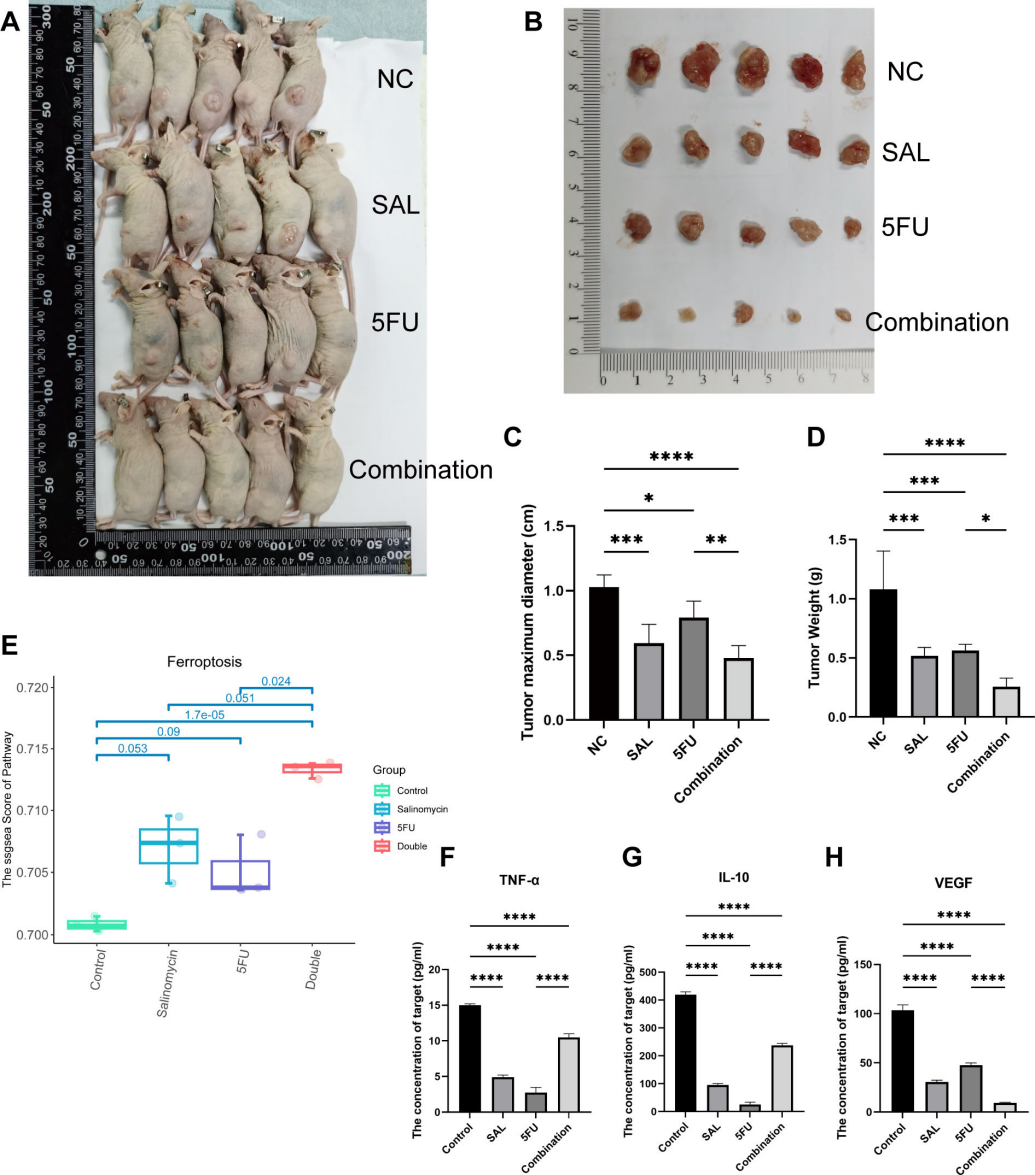
**(A, B)** Different drug groups affect xenograft tumor proliferation in nude mouse tumorigenesis experiments. **(C)** Effects of different drug groups on the maximum diameter of tumors. **(D)** Effects of different drug groups on the weight of tumors. **(E)** Transcriptomics reveals that the drug combination significantly promotes the occurrence of ferroptosis (*p* = 1.7E-05). **(F–H)** Changes in three cytokines in plasma of nude mice in different drug groups. Compared with the control group: * or ns, *p* < 0.05; **p < 0.01; ***p < 0.001; ****p < 0.0001.

As shown in **Figures 5A, B**, nude mouse tumorigenesis experiments were used to confirm the synergistic effect *in vivo*, and the results showed that synergistic use can limit the inhibition of tumor progression: both reducing tumor maximum diameter (*p* < 0.001 for Combination vs. NC) and weight (*p* < 0.0001 for Combination vs. NC, **Figures 5C, D**). In addition, the decreased cytokine of VEGF indicates the decrease of tumor metastasis and invasion ability (*p* < 0.001 for Combination vs. NC, **Figure 5H**). However, the change in TNF-α and IL-10 may mean potentially complex mechanisms of action and changes in the tumor immune microenvironment (**Figures 5F, G**). At the same time, HE staining proved that our different drug groupings did not have adverse effects on the important organs of mice, which means that the dosage of each group is safe (**Supplementary Figure S2**). Collectively, our evidence demonstrates that drug combination therapy can inhibit tumor proliferation *in vivo* as well as more benign biological behavior and biosafety.

### Drug combination between SAL and 5-FU inhibited tumor cell proliferation by activating ferroptosis via downregulating GPX4 and SLC7A11

**Figure 6:**
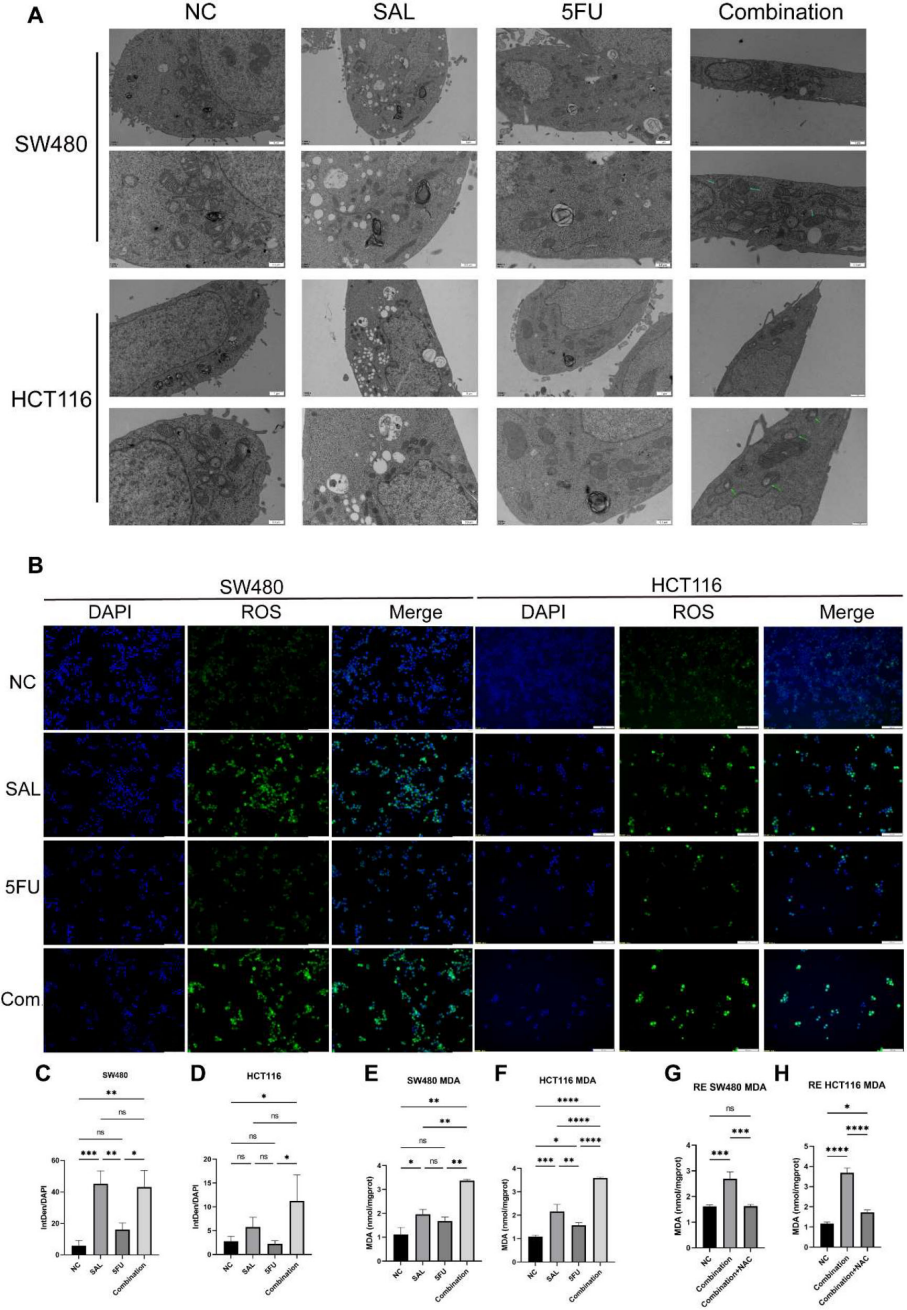
**(A)** Transmission electron microscopy results of different group administration treatments. Among them, more autophagy lysosomes can be seen in both SAL and 5-FU. Ferroptotic mitochondrial changes (as shown by the green arrow) and a small number of autophagy lysosomes can be seen in the combination of the two drugs. **(B)** ROS staining results of SW480 and HCT116 cell lines after treatment with different drugs for 20 h **(C, D)** Statistical analysis of ROS staining results of SW480 and HCT116 cell lines after administration. **(E, F)** Intracellular MDA levels under different group drug treatments of SW480 and HCT116 cell lines. **(G, H)** Synergistically promote an increase in MDA levels, which can be restored by 100 mM NAC in SW480 and HCT116 cell lines. Compared with the control group: * or ns, *p* < 0.05; **p < 0.01; ***p < 0.001; ****p < 0.0001.

We used transcriptome sequencing to investigate specific mechanisms underlying the combination of SAL and 5-FU. Different from the conventional results, we put emphasis on the changes in the level of pathways rather than single genetic perturbation. After scoring by Hallmark (**Supplementary Figure S1**) and common cell death pathways collected by Qin et al. (17), we found that the synergistic effect of SAL and 5-FU promoted the improvement of the ferroptosis pathway (*p* = 1.7E-05, **Figure 5E**), Further research is implemented to confirm it. Electron microscopy results showed that compared with the blank control group, the integration caused some mitochondria to shrink in size, relatively thicken in membrane, and disappear in cristae (shown by the green arrow, **Figure 6A**), confirming that the combination of SAL and 5-FU resulted in ferroptosis. Complementarily, reactive oxygen species (ROS) staining showed that ferroptosis can be induced by SAL alone and in combination with both SW480 and HCT116 (for SW480 Combination vs. NC, *p* < 0.01; for HCT116 Combination vs. NC, *p* < 0.05; **Figures 6B–D**). The levels of MDA in cells assisted to verify this (for SW480 Combination vs. NC, *p* < 0.01; for HCT116 Combination vs. NC, *p* < 0.0001), and these changes could be restored by the ferroptosis inhibitor NAC (100 nM/mL, **Figures 6E–H**): Compared with the control group of 0.1% DMSO, the combination of the two drugs synergistically promotes the increase of MDA levels in the SW480 (*p* < 0.01) and HCT116 (*p* < 0.01) cell lines, and similar changes were also observed compared with the 5-FU-treated group (*p* < 0.01). Meanwhile, after the addition of NAC, the MDA levels significantly promoted by the synergy of two drugs returned to normal for SW480 (*p* < 0.05) and for HCT116 (*p* < 0.001), compared with the combination.

**Figure 7:**
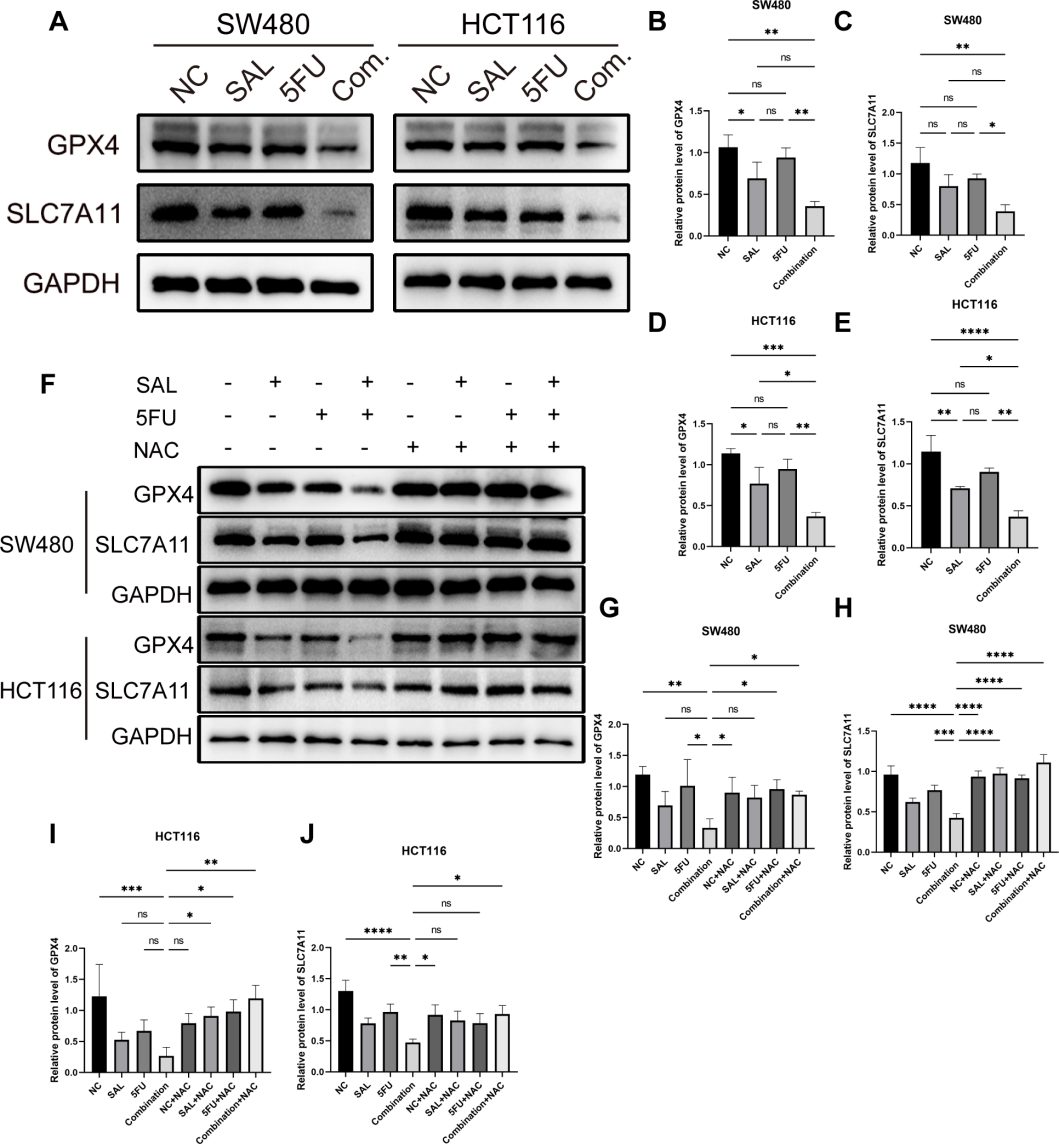
**(A)** Western blot shown the changes in expression levels of key ferroptosis proteins GPX4 and SLC7A11 in SW480 and HCT116 cell lines with different drugs for 20 h **(B–E)** Statistical analysis of corresponding protein, GPX4 and SLC711, of changes of SW480 and HCT116 cell lines after administration. **(F)** The protein expression levels of GPX4 and SLC7A11 that were reduced during the double-drug synergy can be restored to normal levels by 100 mM NAC. **(G–J)** Statistical analysis of counterpart proteins of SW480 and HCT116 cell lines after administration of combination and NAC. Compared with the control group: * or ns, *p* < 0.05; **p < 0.01; ***p < 0.001; ****p < 0.0001.

At the same time, the protein levels of GPX4 and SLC7A11, which were believed to be involved in the clearance of lipid peroxidation by participating in the production and regulation of cysteine, were also observed to decrease, compared with only the 5-FU group (**Figures 7A–E**): for SLC7A11 in SW480 (*p* < 0.01, **Figure 7C**) and HCT116 (*p* < 0.05, **Figure 7E**), and for GPX4 in SW480 (*p* < 0.01, **Figure 7B**) and HCT116 (*p* < 0.01, **Figure 7D**). In parallel, this change could still be restored by 100 nM NAC (**Figures 7F–J**): in SW480 cells, the changes between Combination and Combination + NAC for the protein of SLC7A11 (*p* < 0.0001, **Figure 7H**) and GPX4 (*p* < 0.05, **Figure 7G**) between Combination and Combination plus NAC are statistically significant, as similarly presented in HCT116 cells, for SLC7A11 (*p* < 0.05, **Figure 7J**) and GPX4 (*p* < 0.01, **Figure 7I**).

In short, SAL can induce ferroptosis, consistent with expectations, and further promote the aggravation of ferroptosis by combining with 5-FU. Our study shows that combined use of SAL and 5-FU can greatly promote the expression of ferroptosis by inhibiting.

## Discussion

SAL, a polyether antibiotic, has gained attention for its potential in cancer therapy. Gupta et al. (18), through high-throughput screening of CSC inhibitors, revealed the stem cell inhibitory effects of SAL that can be used to fight Gram-positive bacteria, fungi, parasites, and viruses, among others. Since then, people have begun to continuously explore the anti-tumor effect of SAL and applied it to the treatment of tumors in various systems. Naujokat et al. found in a preclinical study that monotherapy with SAL at doses of 200 to 250 μg/kg significantly benefited several patients with advanced disease who were unresponsive to conventional treatments in 2012. However, one of the patients with advanced squamous cell carcinoma was initially treated with a combination of erlotinib and SAL, but later received SAL alone due to intolerance, and tumor regression was still observed (14). Furthermore, SAL itself or formulations containing SAL have been used to overcome resistance to various chemotherapeutic drugs. For example, Wang et al. and Yue Zhou et al. used SAL in combination with 5-FU (19) and doxorubicin (5) on liver cancer cell lines to reverse resistance to these drugs, respectively. Daman et al. (8) used polymeric micelles loaded with SAL made by polyethylene glycol-b-poly lactic acid to address the gemcitabine resistance on pancreatic cancer cell lines; Mao et al. (9) confirmed that SAL can reverse resistance in cisplatin on the gastric cancer cell line SGC7901/CDDP; Zou et al. (7) demonstrated that SAL can reverse gefitinib resistance on CRC cell lines; Tsakiris et al. (6) proved that SAL administered with B can synergistically promote the growth inhibition of CRC cell lines, achieving varying degrees of the desired effects. Meanwhile, the analogs of SAL, including SAL–isohydroxamic acid conjugates (10), C20-O-alkyl/benzyl oxime derivatives (11), C20-O-acylated analog of SAL (12), and SAL nanocrystals (13), are also used in anti-tumor treatments and have achieved great results recently.

In our study, we successfully found that SAL alone can inhibit the proliferation, migration, and invasion of CRC in a time- and dose-dependent manner. In addition, CCK8 results indicated that various combinations of different concentrations of SAL and 5-FU can synergistically inhibit tumor proliferation. Notably, even the lowest concentration combination of 2.5 μM SAL and 5 μM 5-FU significantly inhibits the proliferation, migration, and invasion of CRC cell lines and increases apoptosis compared to monotherapy with 5 μM 5-FU alone; Wang et al. had the same finding in their work on hepatocellular carcinoma cell lines (19). In Wang et al.’s study, they used SAL and 5-FU at concentrations of 0, 2, 4, 8, and 16 μM, both as monotherapy and as combination therapy. SAL monotherapy significantly inhibited the proliferation of hepatocellular carcinoma cells. Moreover, in the subsequent drug combinations, most combinations showed better inhibitory effects compared to the same-dose 5-FU monotherapy group. Our Annexin-V/PI staining results were consistent with those of Wang: the combination therapy and the 5-FU monotherapy both promoted a higher level of apoptosis. However, to be cautious, further experiments are still needed to validate this part. Meanwhile, fewer number of tumor cells were observed in the Transwell migration and invasion assays in the combination therapy group compared to the 5-FU monotherapy group, and lighter tumor weights *in vivo* were used as additional evidence to support the aforementioned conclusions.

On the other hand, Ebokaiwe et al. (20) and Shen et al. (21), respectively, indicate that SAL can promote T-cell proliferation and macrophage polarization, suggesting the potential value of SAL in modulating immune responses in breast cancer cell lines. Our study indicates that the combination can increase the levels of TNF-α and IL-10, suggesting the potential of SAL to stimulate immune responses in CRC cell lines. However, further experiments are still needed for validation.

The transcriptome results indicate that the ssGSEA score of the ferroptosis pathway captured from KEGG is significantly elevated in the 5-FU monotherapy and control groups, consistent with the findings of Zhou et al. (22) and Chung et al. (7) in CRC cell lines and Antoszczak et al. (23) in pancreatic cancer cell lines. ROS staining and Western blot analysis of the key molecules GPX4 and SLC7A11, along with rescue experiments, further support these conclusions. Under transmission electron microscopy, we observed characteristic mitochondrial morphological changes in both the SAL monotherapy and combination therapy groups: shrunken mitochondria and the disappearance of mitochondrial cristae, consistent with the characteristics of ferroptosis described by Chen et al. (24).

Research indicates that SAL exhibits selective activity against CSCs, which are often responsible for tumor initiation, metastasis, and resistance to conventional therapies. The mechanism behind this cytotoxic effect involves several pathways in CRC; for instance, inhibition of the β-catenin/TCF4E complex blocks the WNT/β-catenin pathway, induces apoptosis activation, and inhibits telomerase activity through a caspase-dependent pathway.

Unfortunately, owing to a lack of experimental design, we did not specifically focus on the changes in the stemness of CRC cell lines, for instance, CD44/CD133. However, studies (19, 25–33) have shown that SAL can indeed inhibit tumor cell proliferation and apoptosis by suppressing cell stemness.

## Conclusions

In summary, the combination of SAL with 5-FU represents a promising approach to inhibiting the development and progression of CRC by effectively targeting both the bulk tumor and the resistant CSC population, thereby enhancing the overall therapeutic efficacy and potentially overcoming drug resistance.

## Data Availability

The original contributions presented in the study are publicly available. This data can be found here: https://www.ncbi.nlm.nih.gov/geo/query/acc.cgi?acc=GSE298808/GSE298808.

## Supplementary material

Supplementary Figure 1
**(A)**: Hallmark signaling pathway ssgsea scoring results; **(B)**: Kegg signaling pathway ssgsea scoring results.

Supplementary Figure 2 HE staining results of important organs involved in tumor formation in nude mice.

Supplementary Figure 3 Statistical analysis of cell cycle distribution in different groups of SW480 and HCT116 cells.

Supplementary Table 1 The IC50 values for HCT116 and SW480 cell lines treated with salinomycin and 5-FU separately for 24, 48, and 72 hours.

Supplementary Table 2 The dose reduction index (DRI) for HCT116 and SW480 cell lines treated with salinomycin and 5-FU.
