# Supplementary material for: Synergistic effects of 5-fluorouracil in combination with salinomycin promoted ferroptosis via inhibiting SLC7A11/GPX4 in colorectal cancer
Source: Front Oncol. 2025 Jun 12;15:1558290. doi: 10.3389/fonc.2025.1558290 (PMC12198170; doi:10.3389/fonc.2025.1558290)
Supplement: Supplementary Figure 1 — (A): Hallmark signaling pathway ssgsea scoring results; (B): Kegg signaling pathway ssgsea scoring results. [file SupplementaryFile1.docx]

**Supplementary materials**


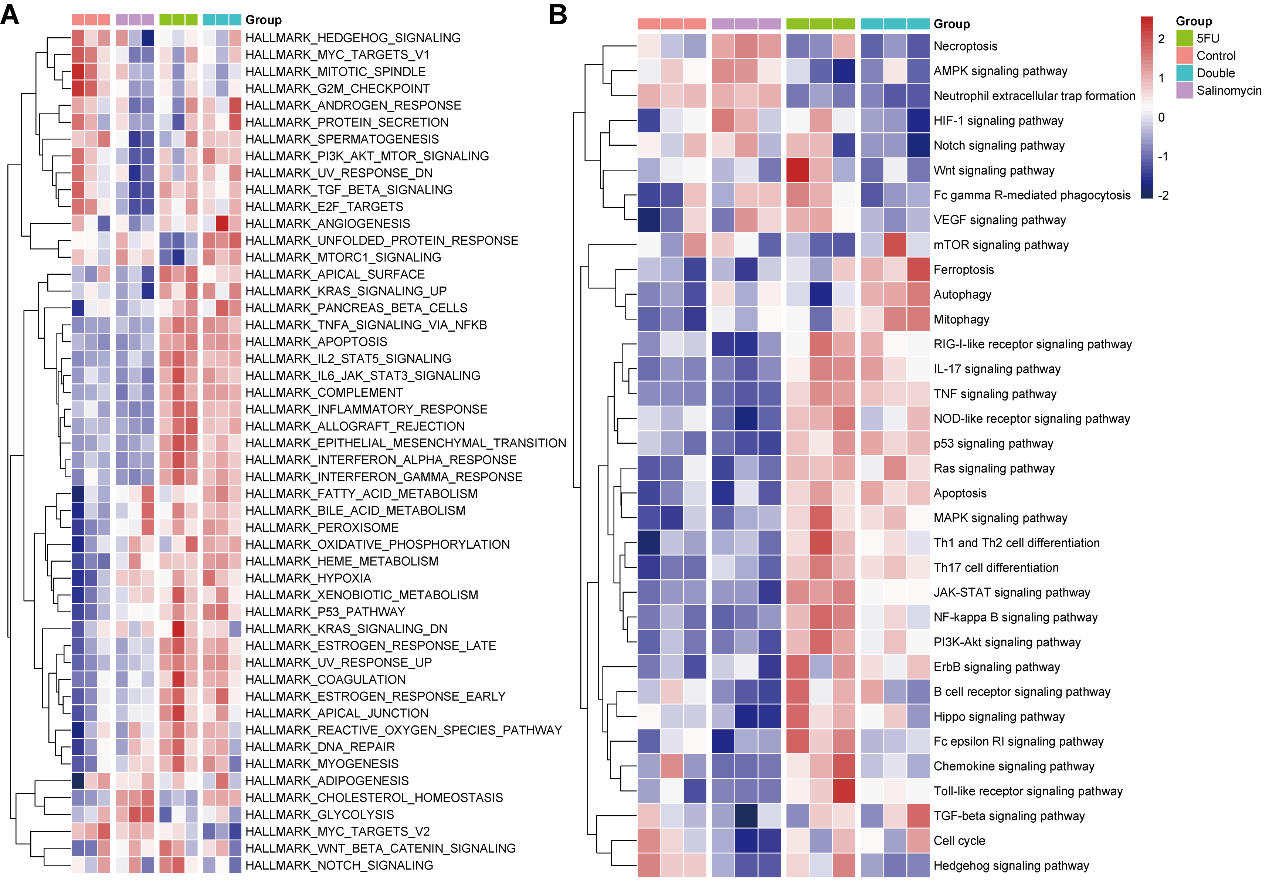


**Supplementary figure 1.** A: Hallmark signaling pathway ssgsea scoring results; B: Kegg signaling pathway ssgsea scoring results


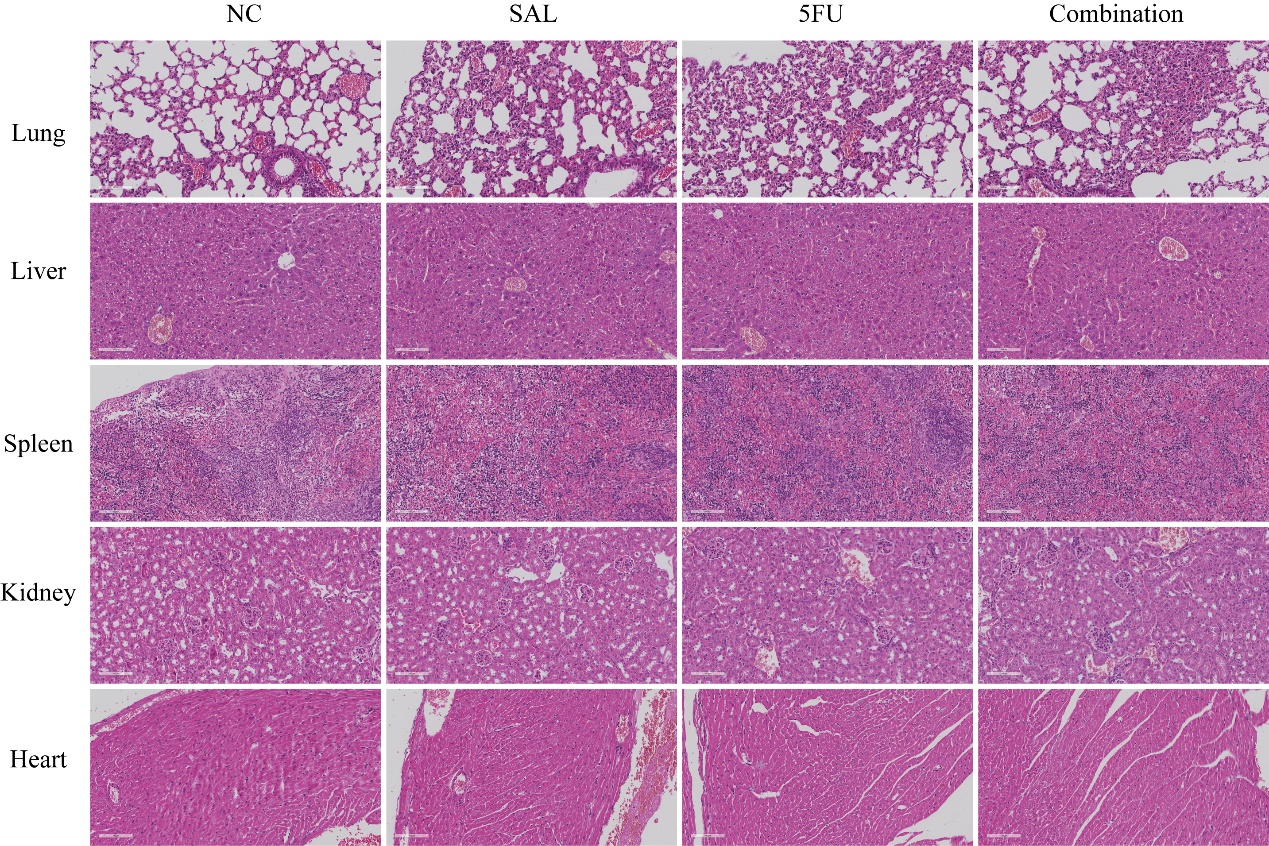


**Supplementary figure 2.** HE staining results of important organs involved in tumor formation in nude mice.


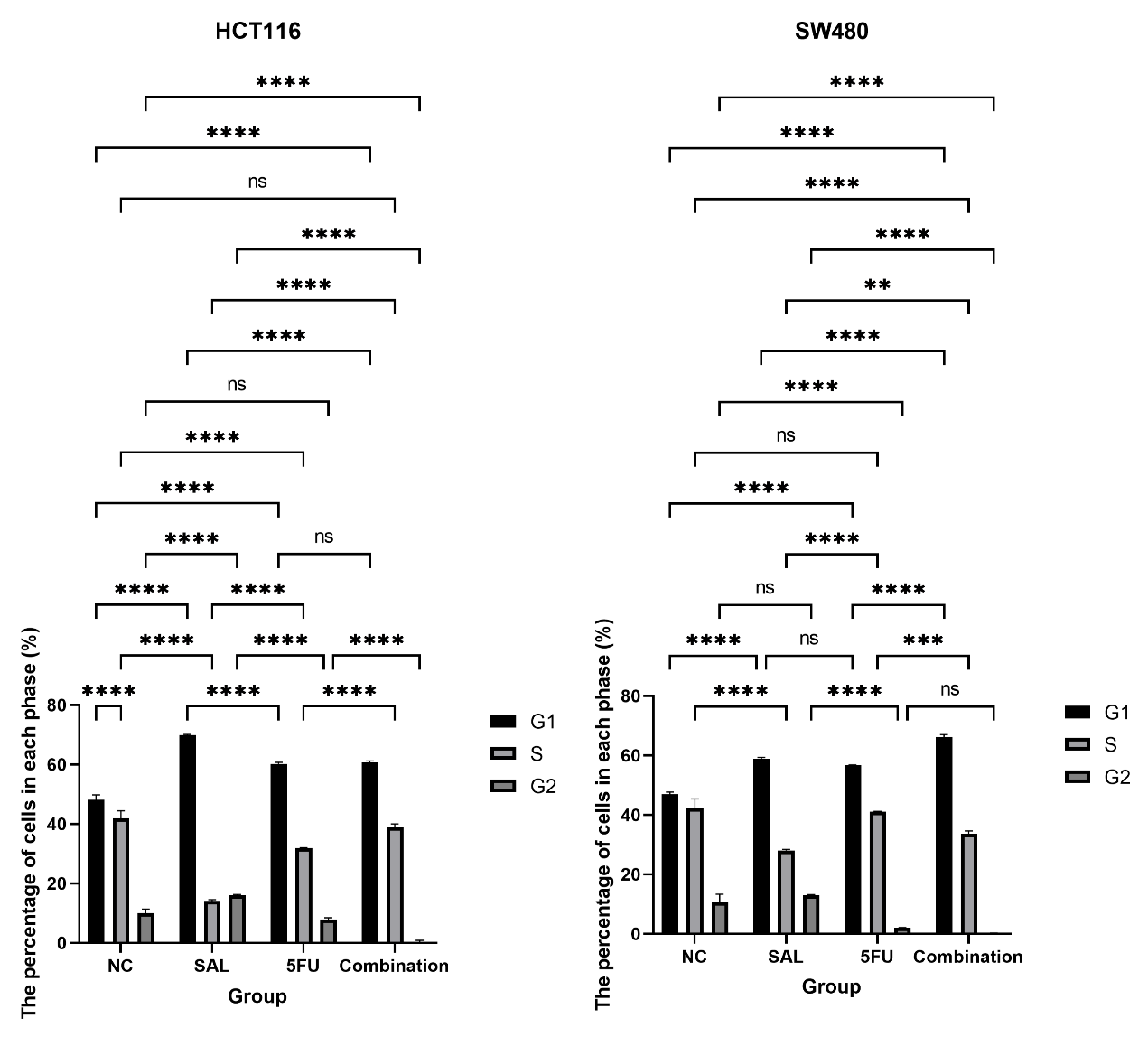


**Supplementary figure 3.** Statistical analysis of cell cycle distribution in different groups of SW480 and HCT116 cells.

**Supplementary Table 1.** **The IC50 values for HCT116 and SW480 cell lines treated with salinomycin and 5-FU separately for 24, 48, and 72 hours.**

| Cancer cell line | IC50 (μM) for SAL | | | IC50 (μM) for 5-FU | | |
| --- | --- | --- | --- | --- | --- | --- |
|  | 24h | 48h | 72h | 24h | 48h | 72h |
| HCT116 | 26.14 | 13.83 | 9.306 | 128.7 | 78.71 | 45.94 |
| SW480 | 19.53 | 17.39 | 14.97 | 109.9 | 81.26 | 35.45 |

**Supplementary Table 2. The dose reduction index (DRI) for HCT116 and SW480 cell lines treated with salinomycin and 5-FU**

| HCT116 | | | | | SW480 | | | | |
| --- | --- | --- | --- | --- | --- | --- | --- | --- | --- |
| Fa | Dose SAL | Dose 5-FU | DRI SAL | DRI 5-FU | Fa | Dose SAL | Dose 5-FU | DRI SAL | DRI 5-FU |
| 0.274 | 8.14396 | 36.0504 | 3.25758 | 7.21007 | 0.299 | 10.7660 | 47.1985 | 4.30640 | 9.43970 |
| 0.345 | 10.7247 | 57.6035 | 4.28987 | 5.76035 | 0.414 | 18.4820 | 102.161 | 7.39279 | 10.2161 |
| 0.428 | 14.3317 | 94.3677 | 5.73266 | 4.71839 | 0.521 | 29.3387 | 197.724 | 11.7355 | 19.7724 |
| 0.525 | 19.7790 | 163.315 | 7.91160 | 4.08288 | 0.573 | 36.7397 | 272.683 | 14.6959 | 6.81707 |
| 0.692 | 35.5329 | 442.797 | 14.2131 | 5.53496 | 0.694 | 64.4485 | 608.730 | 25.7794 | 7.60912 |
| 0.368 | 11.6507 | 66.3262 | 2.33013 | 13.2652 | 0.409 | 18.0778 | 98.9840 | 3.61556 | 19.7968 |
| 0.426 | 14.2352 | 93.2894 | 2.84705 | 9.32894 | 0.478 | 24.4002 | 151.942 | 4.88004 | 15.1942 |
| 0.484 | 17.2722 | 129.664 | 3.45445 | 6.48322 | 0.547 | 32.8131 | 232.014 | 6.56261 | 11.6007 |
| 0.591 | 24.6790 | 238.059 | 4.93581 | 5.95147 | 0.6 | 41.3934 | 323.347 | 8.27867 | 8.08367 |
| 0.755 | 46.1268 | 690.470 | 9.22536 | 8.63088 | 0.769 | 97.2103 | 1095.18 | 19.4421 | 13.6897 |
| 0.468 | 16.3810 | 118.481 | 1.63810 | 23.6961 | 0.479 | 24.5052 | 152.877 | 2.45052 | 30.5754 |
| 0.523 | 19.6485 | 161.484 | 1.96485 | 16.1484 | 0.558 | 34.4144 | 248.361 | 3.44144 | 24.8361 |
| 0.625 | 27.7662 | 290.962 | 2.77662 | 14.5481 | 0.643 | 50.3521 | 427.810 | 5.03521 | 21.3905 |
| 0.655 | 30.9197 | 349.445 | 3.09197 | 8.73612 | 0.742 | 83.1143 | 875.520 | 8.31143 | 21.8880 |
| 0.849 | 75.7906 | 1608.14 | 7.57906 | 20.1018 | 0.921 | 372.083 | 7454.88 | 37.2083 | 93.1860 |
